# Best Practices of Blood Cultures in Low- and Middle-Income Countries

**DOI:** 10.3389/fmed.2019.00131

**Published:** 2019-06-18

**Authors:** Sien Ombelet, Barbara Barbé, Dissou Affolabi, Jean-Baptiste Ronat, Palpouguini Lompo, Octavie Lunguya, Jan Jacobs, Liselotte Hardy

**Affiliations:** ^1^Department of Clinical Sciences, Institute of Tropical Medicine, Antwerp, Belgium; ^2^Department of Microbiology and Immunology, KULeuven, Leuven, Belgium; ^3^Centre National Hospitalier Universitaire—Hubert Koutoucou Maga, Cotonou, Benin; ^4^Médecins Sans Frontières, Operational Center Paris, Paris, France; ^5^Clinical Research Unit of Nanoro, Institut de Recherche en Science de la Santé, Nanoro, Burkina Faso; ^6^National Institute for Biomedical Research, Kinshasa, Democratic Republic of the Congo; ^7^Department of Medical Biology, Cliniques Universitaires, Université de Kinshasa, Kinshasa, Democratic Republic of the Congo

**Keywords:** clinical bacteriology, blood culture, low-resource settings (LRS), laboratory medicine practices, bacteremia diagnosis

## Abstract

Bloodstream infections (BSI) have a substantial impact on morbidity and mortality worldwide. Despite scarcity of data from many low- and middle-income countries (LMICs), there is increasing awareness of the importance of BSI in these countries. For example, it is estimated that the global mortality of non-typhoidal *Salmonella* bloodstream infection in children under 5 already exceeds that of malaria. Reliable and accurate diagnosis of these infections is therefore of utmost importance. Blood cultures are the reference method for diagnosis of BSI. LMICs face many challenges when implementing blood cultures, due to financial, logistical, and infrastructure-related constraints. This review aims to provide an overview of the state-of-the-art of sampling and processing of blood cultures, with emphasis on its use in LMICs. Laboratory processing of blood cultures is relatively straightforward and can be done without the need for expensive and complicated equipment. Automates for incubation and growth monitoring have become the standard in high-income countries (HICs), but they are still too expensive and not sufficiently robust for imminent implementation in most LMICs. Therefore, this review focuses on “manual” methods of blood culture, not involving automated equipment. In manual blood cultures, a bottle consisting of a broth medium supporting bacterial growth is incubated in a normal incubator and inspected daily for signs of growth. The collection of blood for blood culture is a crucial step in the process, as the sensitivity of blood cultures depends on the volume sampled; furthermore, contamination of the blood culture (accidental inoculation of environmental and skin bacteria) can be avoided by appropriate antisepsis. In this review, we give recommendations regarding appropriate blood culture sampling and processing in LMICs. We present feasible methods to detect and speed up growth and discuss some challenges in implementing blood cultures in LMICs, such as the biosafety aspects, supply chain and waste management.

## Introduction

### Scope of This Review

This review provides an overview of current best practices in sampling and processing blood cultures in low- and middle-income countries (LMICs). LMICs are defined depending on gross national income per capita by the World Bank^1^. LMICs face many challenges when implementing laboratory medicine, related to lack of financial and human resources and infrastructure (1). Since most studies on blood cultures have been performed in high-income countries (HICs), many recommendations from these studies cannot be easily adopted in LMICs. This review will therefore focus on blood culture methods and techniques appropriate for settings with limited resources. Studies conducted in LMICs will be mentioned explicitly when available.

Furthermore, attention will be given to caveats and obstacles of implementing blood cultures in LMICs. The details of implementing such a blood culture system in microbial surveillance and techniques used for identification and antibiotic susceptibility testing will not be discussed. In addition, advice on implementation of quality management for clinical bacteriology in LMICs has already been described elsewhere (2).

#### Blood Culture Definitions and Work-Flow

**Figure 1:**
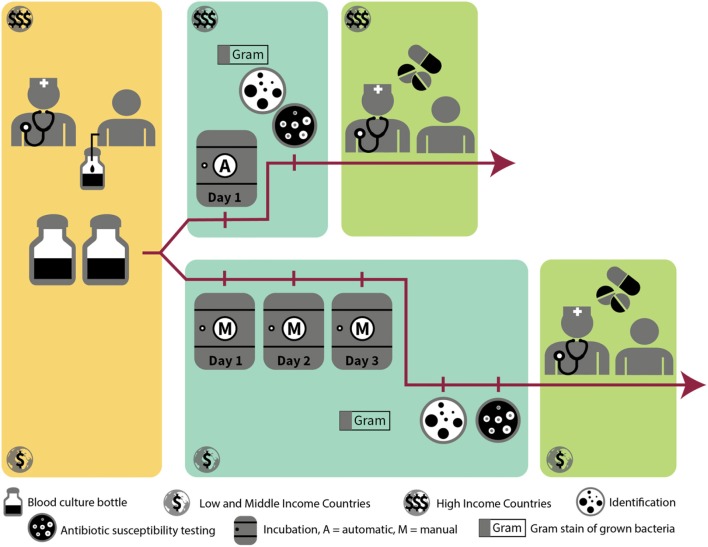
Workflow of grown blood cultures in high-resource vs. low-resource setting.

In normal conditions, blood is sterile. Severe localized or systemic infections can cause micro-organisms to enter the bloodstream through the lymphatic system. This presence of bacteria in the bloodstream is called “bacteremia.” Most of the time, these bacteria are cleared quickly by the immune system. In the case of overwhelming infections or intravascular focus of infection, the immune system may be unable to clear the bacteria from the blood, resulting in a bloodstream infection (BSI) (3). The micro-organisms responsible for this infection can be identified by blood culture. A blood culture consists of a blood sample from a patient, suspected to have a BSI, which is inoculated into a specialized blood culture bottle containing a broth (i.e., liquid) medium that supports optimal growth of bacteria. The concentration of bacteria in the blood of patients with BSI is very low (4), therefore direct culture on an agar plate cannot detect the presence of bacteria in the patient's blood. Once the blood is inoculated into the blood culture bottle, further amplification of the bacteria can take place, ultimately leading to visible bacterial growth. When growth of bacteria is detected in the blood culture bottle, a Gram stain of the blood-broth mixture is done to confirm presence of micro-organisms and distinguish between Gram-positive, Gram-negative bacteria and yeasts (Table 1). This and other microscopic information, such as shape and configuration of the bacteria, can orient the clinician to the identification of the bacteria causing the BSI and hence to the most appropriate antibiotic treatment. A subculture of the blood-broth mixture on an agar plate is then done to obtain colonies of the pathogen, on which further identification and antibiotic susceptibility testing can be performed. See Figure 1 for a visualization of the blood culture workflow and Table 2 for definitions regarding blood cultures.

**Table 1:** Examples of common bacterial species grown in blood cultures.

	**Gram-positive**		**Gram-negative**		**Yeast**
	**Pathogen**	**Contaminant**	**Pathogen**	**Contaminant**	**Pathogen**
Aerobic		*Bacillus* species	*Pseudomonas aeruginosa*	Stenotrophomonas maltophilia^*^	*Cryptococcus neoformans*
			*Burkholderia pseudomallei*	*Pseudomonas* species (non-aeruginosa)^*^	
			*Acinetobacter* species		
Anaerobic	*Clostridium* species	*Cutibacterium acnes*	*Bacteroides* species		
Facultative /aero-tolerant	*Streptococcus pneumoniae*	Coagulase-negative *Staphylococcus* spp.	*Escherichia coli*		*Candida albicans*
	*Staphylococcus aureus*	*Micrococcus* species	*Klebsiella pneumoniae*		*Candida glabrata*
			Non-typhoidal *Salmonella*		
			*Salmonella* Typhi		

* *Uncertainty of interpretation according to current literature*.

**Table 2:** Definitions used in or related to the blood culture process.

Automated blood culture system	Blood culture system that uses equipment (an automated incubator) for incubation, agitation, and monitoring of blood culture bottles for microbial growth
Bacteremia	Presence of bacteria in the bloodstream
Biphasic blood culture system	Blood culture system in which a single bottle consists of a liquid broth phase and a solid agar phase; designed so the agar can be irrigated (and inoculated) with the broth medium
Blind subculture	Subculture performed from the blood culture bottle in the absence of visual signs of growth
Blood culture	Specimen of blood sampled through 1 venipuncture (possibly divided into multiple blood culture bottles) for the culture of micro-organisms
Blood culture broth	A liquid enrichment medium for the growth of bacteria used in the diagnosis of BSI. Usually contains peptides of animal origin and dextrose.
Bloodstream infection	Infection with the presence of bacteria in the blood
Bloodstream infection episode	A BSI episode is defined as (1) the initial recovery of a pathogen in a blood culture, (2) the recovery of a pathogen different from the initial pathogen ≥48 h after the recovery of the initial pathogen, or (3) the recovery of the same pathogen after at least a 14-day interval since the previous grown culture with this pathogen (5, 6)
Contamination	Growth of a micro-organisms in a blood culture that was introduced into the culture during blood culture collection or processing and that is not the cause of the suspected BSI
Culture medium	Substance used to facilitate growth of bacteria; can be solid (agar) or liquid (broth)
Endocarditis	Infection of the inner layer of the heart, the endocardium, often involving the heart valves
Fastidious organisms	Organisms that require special nutritional and incubation conditions for culture (e.g., addition of certain nutrients, incubation in carbon dioxide atmosphere)
HACEK organisms	A group of Gram-negative bacteria that are unusual causes of endocarditis; consists of the following species: *Haemophilus parainfluenzae, Haemophilus aphrophilus, Actinobacillus actinomycetemcomitans, Cardiobacterium hominis, Eikenella corrodens*, and *Kingella kingae* (7)
Manual blood culture bottle	Blood culture bottle that is designed for use in a manual blood culture system, i.e., without using automated equipment
Manual blood culture system	Blood culture system that processes blood culture bottles without the use of automated equipment
Non-fermenting Gram-negative organisms (non-fermenters)	Heterogenous group of Gram-negative bacilli that are aerobic and cannot metabolize carbohydrates through fermentation; mainly implicated in healthcare-associated infections and often resistant to many types of antibiotics
Subculture	A secondary culture of bacteria made from material derived from another culture, such as the blood-broth mixture of a blood culture bottle or the colonies on an agar plate
Terminal subculture	A subculture done at the end of the incubation period of blood culture bottles that failed to show signs of growth, to confirm the absence of growth of micro-organisms

#### Indications for Blood Culture

Blood cultures must be obtained whenever there is a clinical suspicion of BSI. However, it is currently not clear which clinical signs are good predictors for BSI. Many predictive models to optimize the yield of blood cultures have been proposed, but so far only two of these have been shown to reliably distinguish between high (>30%) and low (<3%) risk of BSI, according to a 2015 systematic review (8). Of these, one model is a computerized system using a causal probabilistic network with input of many different variables (9), compromising its possible use in LMICs, and the other has been validated only for patients with community-acquired pneumonia (10). Moreover, none of these validated models are used in routine clinical practice, possibly because they are too complicated to calculate at the bedside and because of their reliance on laboratory data that are not available at the time of blood sampling (8).

**Figure 2:**
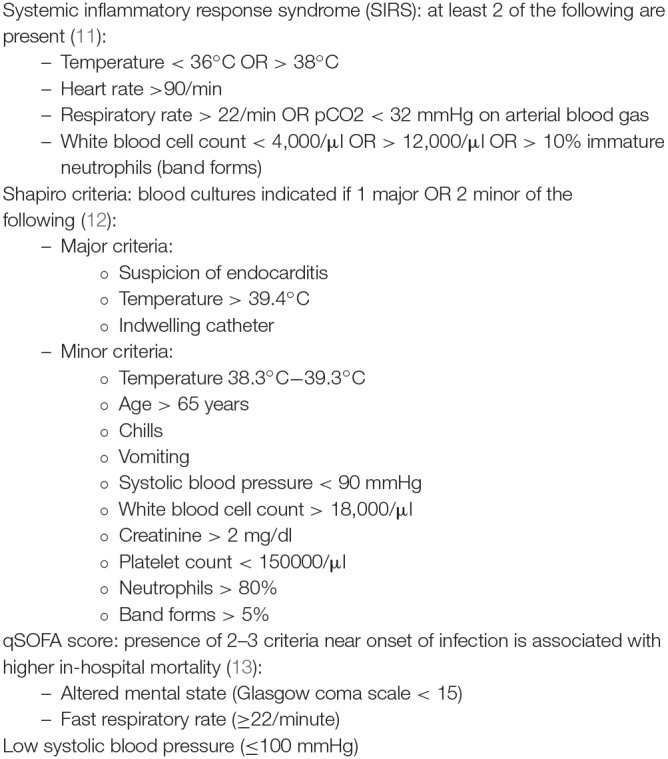
Models and scores to predict BSI: SIRS criteria, Shapiro criteria & qSOFA score (11–13).

Other well-studied clinical predictive models are the SIRS criteria (systemic inflammatory response syndrome) and the Shapiro criteria (11, 12) (see Figure 2). Both these models have demonstrated high sensitivity but low specificity (14–16) and rely on laboratory parameters that are not readily available in most LMICs. Their usefulness in daily practice is therefore rather limited.

In 2016, the Third International Consensus Definitions Task force described an easy clinical score to identify patients at risk of sepsis, the quick Sequential Organ Failure Assessment (qSOFA) score (13) (see Figure 2). Sepsis is defined as life-threatening organ dysfunction caused by a dysregulated host response to infection (17). It can occur with or without bloodstream infection. The predictive validity of the qSOFA for in-hospital mortality outside of intensive care units was higher than that of the SOFA and SIRS criteria (which are more elaborate). However, as qSOFA was validated on patients already suspected of infection, it cannot be used to differentiate patients with infection from those without infection.

**Figure 3:**
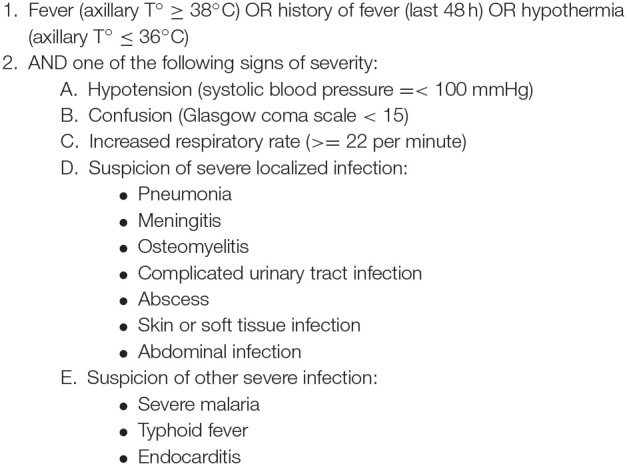
Proposal for clinical indications of bloodstream infections in LMICs (3, 17–19).

In Figure 3, we propose a set of clinical indications for sampling blood cultures in LMICs. These indications were based on known common causes of BSI (3), the qSOFA criteria (13) and experience within our research network (18, 19). For neonates, blood culture indications are different and more complex; we refer to other resources for more information (20–22).

#### Collection of Blood for Blood Culture

The process of collection of blood for blood culture is very important for the quality of the results (23). It requires applying a tourniquet on the arm, palpating the vein that will be used for the sampling, and applying appropriate antiseptics at the place of sampling. After antisepsis, the vein should not be touched anymore unless wearing sterile gloves. Next, the vein is pierced with either a needle and syringe or a butterfly needle. A sufficient volume of blood is aspirated either directly into the blood culture bottle (with certain bottle types and use of butterfly needle) or into a syringe and next divided over the blood culture bottles.

#### Automated vs. Manual Blood Cultures

Further processing of cultures is described in this text with an emphasis on so-called “manual” blood culture systems (i.e., not making use of automated equipment). These systems rely on the use of appropriate blood culture bottles, which are placed in a conventional static incubator and are inspected daily to visually detect signs of growth of micro-organisms. This contrasts with the automated systems, which over the past decades have largely replaced manual blood culture systems in HICs and are the current standard (23). In these systems, the carbon dioxide production by micro-organisms in the bottles is continuously monitored, either by colorimetric or fluorescent detection. During incubation, the bottles are continuously agitated in the automated equipment. The current automated systems show better performance than manual systems in terms of yield and especially speed of growth, as recent studies from LMICs such as Egypt, Pakistan, and India have shown (24–26). However, these automated systems are costly, require regular maintenance and are not adapted to tropical, dusty environments, impeding the sustainable implementation of this technique in many LMICs outside of study sites, large reference laboratories or private laboratories in the capital cities (1, 27). Therefore, manual blood culture bottles are still most frequently used in LMICs and contribute worldwide to approximately the double of market share compared to automated systems (27). These manual blood culture bottles will be discussed and evaluated keeping the World Health Organization's (WHO) ASSURED criteria for diagnostics in LMICs in mind; i.e., they must be affordable, sensitive, specific, user-friendly, robust, rapid, equipment-free, and delivered to those who need it (28). Recently, the same research group proposed to add two criteria to this list: the REASSURED criteria put an additional emphasis on real-time connectivity and ease of specimen collection (29).

### The Importance of Blood Cultures in LMICs

BSI presents a high burden of morbidity and mortality worldwide, with the highest burden in neonates and children (30). Exact figures for incidence and associated mortality of BSI are scarce to non-existent in many LMICs, because of a lack of bacteriological laboratories and surveillance (31–34). Even in HICs, however, mortality of BSI is still substantial, ranging between 17 and 29% (31, 32, 35, 36). Key interventions to decrease mortality of BSI are sampling of blood cultures before administration of antimicrobial therapy and daily reassessment of antimicrobial therapy for optimization and de-escalation, based on the identification and antibiotic susceptibility testing of the pathogen (37). As survival of BSIs is inversely related with time to adequate antibiotic therapy (38), it is also important that results of blood cultures are available as soon as possible.

Historically, the default acute fever diagnosis in tropical settings has been malaria, which still represents a major childhood killer. Based on symptoms and clinical presentation, severe malaria is indistinguishable from BSI, contributing to overdiagnosis of malaria and underdiagnosis of BSI particularly in children <5 years old (39, 40). Moreover, morbidity and mortality of malaria have been declining steadily over the last few decades. The number of malaria deaths globally fell from an estimated 839,000 in 2000, to 445,000 in 2016, which is a decline of 47% (WHO World Malaria Report 2017^2^. In contrast, progress in outcomes for sepsis has been much more modest, with a decline in mortality from sepsis of only 25% between 2000 and 2015—moving it upwards in the rank of deadly diseases requiring pathogen-based diagnosis (41). Increasing evidence of co-infection of malaria with non-typhoidal *Salmonella* (42, 43) further emphasizes the importance of blood culture in these settings.

The spectrum and frequency ranking of bacteria causing BSI in LMICs differ from those recorded in HICs. Pathogens like *Salmonella enterica* or *Burkholderia pseudomallei* are uncommon in HICs but account for a large proportion of pathogens in Africa and South-East Asia (5, 19, 44–47). Typical childhood pathogens for which vaccination is available in HICs, such as *Haemophilus influenzae* and *Streptococcus pneumoniae*, are also more common in LMICs (45, 46).

### Feasibility of Blood Cultures in LMIC Settings

In the majority of blood cultures only one organism grows. Polymicrobial infections make up 6–18% of all BSI episodes, with higher proportions of polymicrobial infections seen in patients with chronic conditions, malignancies and nosocomial infections (48–54). Polymicrobial infections are probably less prevalent in LMICs, because of differences in patient population. A 2010 review of BSI in Africa found only 1.2% of BSI episodes to be polymicrobial (45). An analysis of BSI episodes in Cambodia showed that only 4.9% of episodes were polymicrobial (5). A convenient consequence of this is that the work-up of blood cultures for identification and antibiotic susceptibility testing is relatively straightforward, as no further isolation of different possible pathogens has to be done. In comparison to other specimens, it is usually easy to differentiate between pathogens and contaminants, and correct identification of the most common causative pathogens can be achieved with simplified techniques. Indications for blood cultures are simple to standardize hospital-wide, and quality indicators to monitor compliance to procedures are more clearly defined than for other specimens (1).

For these reasons, blood culture is an easy first step in starting clinical bacteriology in any laboratory, and it is recognized as a priority specimen for surveillance of antimicrobial resistance by WHO because of its clinical importance and the accurate and uncomplicated methods of detection (55).

### Importance of Blood Culture for Antibiotic Stewardship and Hospital Infection Prevention and Control

Because of the severity of most BSIs, blood cultures have high clinical relevance. Rapid notification of even preliminary blood culture results, such as the result of a Gram stain of a grown culture, has been shown to have a large impact on rational antibiotic prescriptions, length of hospitalization and even patient survival (56–62).

Moreover, results from blood culture surveillance can be efficiently used in infection prevention and control. Analysis of trends per hospital ward can alert outbreaks (63, 64). Moreover, blood culture results allow to make the distinction between healthcare-associated and community-acquired infections. The WHO defines a healthcare-associated infection as “an infection occurring in a patient during the process of care in a hospital or other health-care facility which was not present or incubating at the time of admission” (65). This term has replaced the term “nosocomial” or “hospital-acquired” infection, as it was acknowledged that the epidemiological and microbiological features of infections acquired in the hospital were very similar to those acquired in other types of healthcare facilities (65–68). A healthcare-associated infection is thus defined as an infection that presents itself more than 48 h after the patient has been admitted to the hospital, or within 48 h of admission if the patient received care at any other facility (including at home) before the hospital admission (66).

However, specialized home care and referrals from other healthcare facilities are rare in LMICs and usually healthcare-associated infections in these settings can accurately be defined as infections with a first positive culture obtained at least 48 h after the moment of admission (similar to the previous definition for hospital-acquired infections). This definition is also used in the Global Antimicrobial Resistance Surveillance System (GLASS) report for use in surveillance of healthcare-associated infections (55).

Monitoring and surveillance of blood culture results can shed a light on the type, impact and number of healthcare-associated infections in a facility. This information can guide selection of appropriate prevention and control measures.

## Methods and Techniques for Processing Manual Blood Cultures

Despite continuous advances in molecular techniques and biomarkers, blood culture remains the gold standard for diagnosis of BSI (23). As mentioned above, LMICs still largely rely on manual blood culture systems, because of the financial and logistic challenges associated with automated systems.

Manual blood cultures are usually incubated for 7 days at 35°C (69, 70). The incremental value of bacterial growth on day 6 and 7 may be limited; a study from 1985 showed that 89% of isolates had been recovered by day 5; many of the isolates retrieved on day 6 and 7 were probable contaminants (71). This observation holds true even for fastidious organisms, such as the HACEK group bacteria (see Table 1 for definitions) (72). As local differences in pathogens can impact the effect of a shorter incubation, the guidelines of the American Society for Microbiology (ASM) recommended examining the impact of a 5-day instead of a 7-day incubation per site and described a procedure to do so in its 2004 edition (73).

For automated blood culture systems, incubation of 5 days has been shown to be sufficient, even for fastidious organisms such as members of the HACEK group (endocarditis) and *Brucella* species (72, 74) (see Table 1). For most pathogens, incubation of 3 days would already suffice (75).

### Broth Type and Additives to Promote Growth in Blood Cultures

#### Broth Medium

The most important feature of a blood culture bottle is that it should adequately support bacterial growth. For this purpose, a variety of peptide broths and additives are available (see Table 3). There is no single “optimal” broth medium; most broths contain dextrose and animal-derived complex peptide molecules. Broth media which support growth of a wide range of bacterial species include tryptic soy broth, also known as soybean-casein digest broth, supplemented peptone broth and brain heart infusion broth. Other commonly used broths such as thioglycolate broth, thiol broth, Columbia and Brucella (hypertonic) broth are also adequate for bacterial recovery (69, 88). For anaerobic bacteria, Columbia broth, pre-reduced peptone broths, thioglycolate broths, and thiol broths are advantageous, supposedly based on their low redox potential (76, 77, 89–91).

**Table 3:** Media composition of manual blood culture bottles.

**Medium or Component/Additives**	**Short description/Comment/References**
**CULTURE MEDIUM**	
Thioglycolate	Favors growth of anaerobes (76)
Tryptic Soy broth (TSB)	General purpose medium, favors *Pseudomonas* species (77, 78)
Thiol broth	Favors growth of *Streptococcus* species (78)
Brain-Heart Infusion (BHI)	General purpose medium, facilitates recovery of yeasts and Gram-positive organisms (79)
Columbia broth	General purpose medium, favors growth of anaerobes
Supplemented peptone broth	General purpose medium; superior to TSB for most common pathogens in blood culture (80)
Hypertonic medium (Brucella broth)	Supposedly improves cellular stability and increases recovery rates of some bacteria, including *Staphylococcus aureus, Escherichia coli, Candida species* (81); evidence regarding its efficacy is mixed (77)
**ADDITIVES**	
Sodium-polyanethole sulfate (SPS)	Anticoagulant; SPS also inhibits lysozyme, inactivates clinically achievable concentrations of some aminoglycoside and polymyxin antibiotics, inhibits parts of the complement cascade, and inhibits phagocytosis (69). Higher SPS concentrations, while promoting the recovery of Gram-positive cocci, decrease the recovery of Gram-negative bacteria. Certain species of bacteria are inhibited by SPS, such as *Neisseria* species, *Peptostreptococcus anaerobius, Moraxella catarrhalis*, and *Gardnerella vaginalis* (69, 77).
Gelatin	Counteracts the inhibition of growth of bacterial species by SPS *in-vitro* (80, 82). Evidence for its clinical efficacy however is not strong (82–86).
Yeast extract	Promotes bacterial growth (77)
Saponin	Lytic agent (used in lysis-centrifugation system); improves recovery of *Streptococcus* species
Hemin (X-factor)	Promotes growth of fastidious organisms such as *Haemophilus influenzae* and *Neisseria* species (87)
NAD (V-factor)	Promotes growth of fastidious organisms such as *Haemophilus influenzae* and *Neisseria* species (87)
Pyridoxine	Promotes growth of pyridoxine-dependent organisms such as certain *Streptococcus* species
Para-amino benzoic acid	Inhibits the effect of sulfonamide antibiotics
Cysteine	Reducing agent; improves recovery of anaerobic bacteria and *Streptococcus pneumoniae* (77)

Regarding the volume of broth, a blood-broth ratio of 1:5 to 1:10 should be respected to optimize growth (92). In children, higher blood-broth ratios (e.g., 1:50 to 1:100) are acceptable (93).

#### Additives to Promote Growth

Supplementation of the broth medium can further promote growth. A number of additives have been defined as growth enhancers in blood cultures, but the effect of additives is limited to the organism targeted for growth (see Table 3).

One of the most important additives is sodium polyanethole sulfonate (SPS). This is an anticoagulant, which in addition has been shown to have a stabilizing effect on microbial growth in blood cultures (69, 88). The typical concentration of SPS ranges from 0.025 to 0.05% (3, 77, 88, 94). The utilization of SPS has greatly reduced time-to-detection for many bacteria, and it is generally agreed to be an indispensable component of blood cultures.

Another commonly used additive is saponin, which is added in some blood culture systems as a lytic agent with subsequent centrifugation (Isolator system from Abbott Laboratories, Chicago, USA). A 1998 study also showed an increased recovery rate with shorter time-to-detection with the addition of saponin to the blood culture media of an automated system (95). Saponin is also widely used in anaerobic bottles in the BACTEC automated blood culture system, in combination with resin-supplemented aerobic bottles (96)^3^.

#### Resins and Charcoal

All guidelines on sepsis management stress the importance of sampling blood for blood culture before administration of antibiotics (37). The negative impact of antibiotic use prior to sampling on the yield of blood cultures is substantial, as the presence of antibiotics in the serum can inhibit the growth of bacteria (97–100). However, patients in LMICs are often already under antibiotic treatment before presenting to the hospital (101), since antibiotics are often readily available over-the-counter in these settings (102–104). To counteract the effect of antibiotics on growth of bacteria in the broth, antibiotic removal devices have been in use since 1982 (105). Nowadays, this role is largely taken up by resins.

Antimicrobial-binding resins and charcoal are generally considered effective in increasing microbial recovery rates. More microorganisms, particularly staphylococci and yeasts, are recovered from formulations with these additives, compared to non-supplemented formulations (106–109). There is no firm clinical evidence that the increased microbial recovery rates are due to inhibition or removal of antibiotic substances, although *in-vitro* research has extensively shown this to be the case (69, 110–113). Most resins are formulated as highly porous polyvinyl and benzene within a spherical bead; there are cationic ion-exchange resins and polymeric adsorbent resins (77). In addition to binding of antimicrobial agents, resin beads provide additional surface area for bacterial growth and help filter and bind components of the complement cascade (69). The exact formulations and balance between ratios of nutrients and resins is often proprietary and thus unknown.

The drawbacks of adding resins to a blood culture medium are the non-specific neutralizing and binding properties of the resins that can result in the removal of nutrients and other substances required for bacterial growth. Moreover, addition of resins to the blood culture broth changes the medium's dynamics, shortens its shelf life and impacts the visual reading of growth in the bottles (90). The addition of resins may even increase the time-to-detection for some bacteria, such as *Pseudomonas* species (113).

However, in automated systems, resin-supplemented broths are superior to general broths and broths containing charcoal (114–118). Importantly, resins also do not interfere as much with Gram staining and reading as does charcoal (114).

#### Gas Phase of the Bottle

For aerobic blood culture bottles, the headspace contains ambient atmosphere to which different amounts (5–10%) of carbon dioxide (CO_2_) have been added. For anaerobic blood culture bottles, headspace contains CO_2_ and nitrogen dioxide (NO_2_). Actual amounts of CO_2_, O_2_ and redox potential vary widely, mostly depending on the manufacturing practices of the blood culture bottles (90).

A portion of the headspace atmosphere is evacuated to create a partial vacuum; thus, blood culture bottles contain an atmosphere in the bottle headspace that has a lower pressure than the atmosphere, enabling easy sampling (88). Not all commercially available bottles contain a vacuum; depending on the sampling techniques, this can create problems during sampling.

#### Detection of Mycobacterial and Fungal Infections

For detection of mycobacterial, fungal or yeast infections in the blood, other broth types and incubation times may be needed. For detection of yeast, such as *Candida* species, regular blood culture bottles and incubation times are advised (74), although *Candida* has a slightly longer time-to-detection than most bacteria (119, 120). Specialized bottles for detection of fungi in blood exist and are more effective than regular aerobic blood culture bottles (121–123), but are not routinely used.

In case of suspicion of invasive filamentous or dimorphic fungi infection, such as *Histoplasma* spp., the use of lysis-centrifugator tubes (Isolator system, Abbott Laboratories) is advised (74, 124). In this method, blood cells are lysed by saponin and the sample is centrifuged. The resulting sediment is then cultured directly on blood agar (69). Lysis-centrifugation can also be used for detection of bacteria, for which a study from India showed lower sensitivity but shorter time-to-detection than conventional blood culture using trypticase soy broth (125). Older studies had already shown good results for detection of bacterial or fungal BSI, but with the drawback of higher contamination rates (126, 127). Moreover, it is more time-consuming than conventional blood culture.

For mycobacteria, lysis-centrifugation has also been used to good effect, including in some LMICs (128, 129). Currently, the use of specific bottles for mycobacteria is advised (74). Longer incubation times are necessary for both mycobacterial infections and filamentous fungal infections (74).

### Visual Detection

#### Visual Signs of Growth

**Figure 4:**
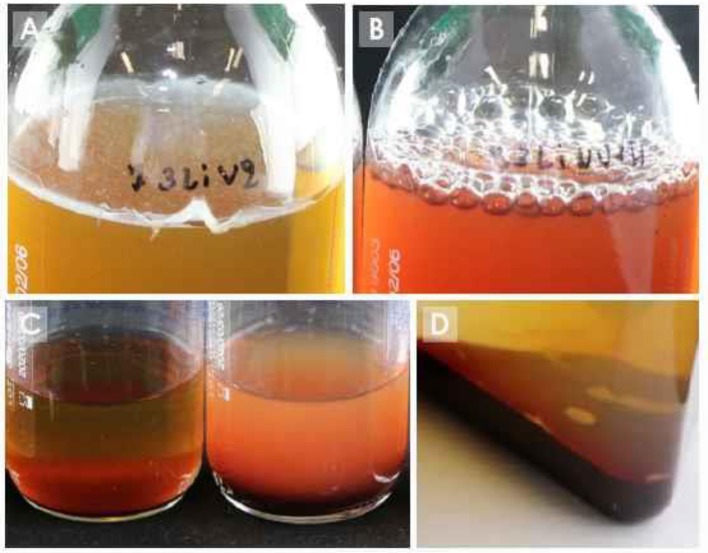
Signs of growth in blood culture bottles. **(A)** pellicle formation on surface; **(B)** gas production; **(C)** turbidity (left bottle: no growth; right bottle: turbidity); **(D)** puff balls.

Growth detection of manual blood culture bottles depends on visible changes in the broth such as turbidity, hemolysis, puff balls, and gas production (see Figure 4). Inspection of the blood culture bottles is done by the laboratory technicians with the naked eye. Therefore, the blood culture bottle must be designed to optimize this visual detection. For example, see-through plastic or glass must be used. The addition of resins or charcoal, described above, can create a haziness in the broth, compromising visual detection of growth (90), while addition of charcoal renders visual detection of growth nearly impossible due to the dark coloration of the broth.

#### Biphasic Bottles

**Figure 5:**
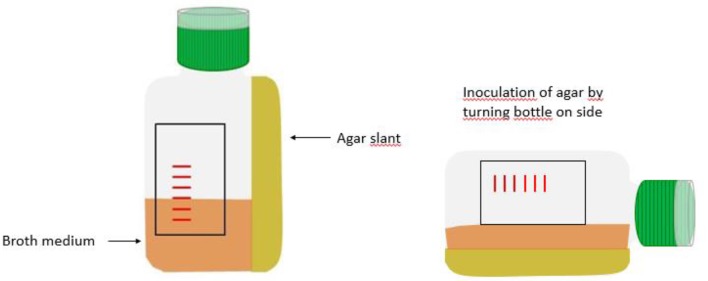
Schematic representation of biphasic bottle.

Biphasic bottles can be an option to facilitate growth detection. They consist of a liquid phase (the broth) and a solid phase in the form of an agar slant (see Figure 5). These bottles were first developed by Castañeda in 1947 for the isolation of *Brucella* (130). *Brucella* species grow slowly with a need for extended incubation times. Because of hemolysis and debris of dying red blood cells, turbidity can no longer be reliably assessed beyond 7 days and repeated subcultures are needed to detect growth. By obviating the need for frequent subculture and its associated infection risks for the laboratory staff handling the blood cultures, biphasic bottles are useful for the isolation of this pathogen. Biphasic media also facilitate the detection of growth of other bacteria for non-expert users, as growth of colonies on the agar may be easier to detect than subtle changes in the broth. Additionally, there is the theoretical advantage of bypassing the initial subculture step in the process of identification and antibiotic susceptibility testing when using the colonies growing on the agar slant, resulting in a potential 18–24 h reduction of the turnaround time in comparison with the conventional blood culture bottles (131).

**Figure 6:**
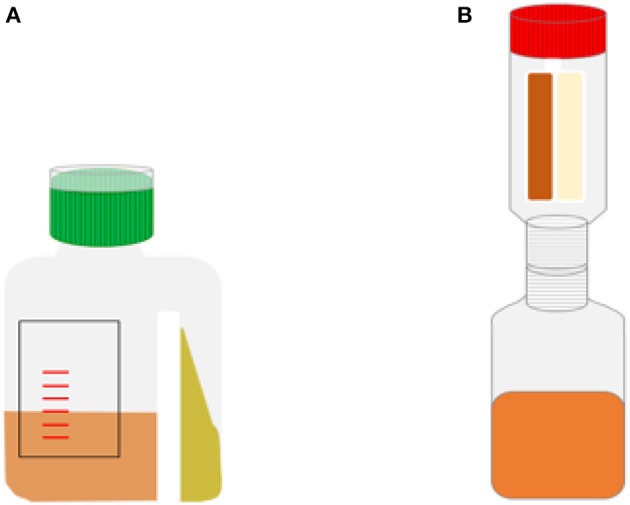
Schematic representations of special biphasic bottle designs. **(A)** bottle used in study by Weckbach et al. (131). **(B)** Septi-Chek system.

Published literature on the effectiveness of biphasic blood culture bottles for bacteria other than *Brucella* is scarce and there are no recent studies (131–136). Overall, those studies indicate superior performance of biphasic bottles for Gram-positives in comparison to other, non-biphasic, manual blood culture bottles. With regards to the speed of growth, a study by Brook et al. showed slower growth in the biphasic bottle as compared to a monophasic bottle (135). Weckbach et al. used a special biphasic bottle design (Figure 6A), physically separating the agar from the broth, and found faster recovery of yeasts (*Candida* spp.) and *Pseudomonas* spp. with the biphasic bottle compared to the conventional bottle. Because of easy subculture, isolated colonies were also available much sooner with the biphasic bottle compared to subculture from the regular bottle (131). Some other studies also found that detection of growth on the agar slant preceded detection of growth in the broth (134, 137). However, the few studies done suffer from sample sizes and a lack of comparability which precludes sound conclusions on the advantages of the biphasic blood culture bottles.

A variation to this type of biphasic blood culture bottle is the connection of a cylindrical “paddle,” containing solid agar plates, to a blood culture bottle, effectively separating the agar and broth phase (Figure 6B). Subculturing can then be done by inverting the bottle and flooding the agar plates with the broth-blood mixture. This can be done at various intervals to optimize isolation. A well-known example of this type of bottle was the Septi-Chek blood culture system by Becton-Dickinson Diagnostics (Franklin Lakes, New Jersey, United States), which was based on a conventional blood culture bottle to which a transparent plastic chamber was connected, which contained a panel of three solid agar media allowing to visualize growth and preliminary identification. This system had promising results and was widely used, but it is no longer commercially available (138–141).

### Volume of Blood to Be Sampled

#### Low Concentration of Bacteria in Blood Necessitates Large Volume

The concentration of micro-organisms in blood of patients with BSI ranges between <1 colony forming unit (CFU)/ml to 10 CFU/ml of blood in adults (4). To avoid missing BSI with a low bacterial load in the process of culturing blood, it is recommended to sample as large a volume as possible for culture. Multiple studies have already shown that higher volumes of cultured blood lead to higher rates of detection (6, 142–147). Therefore, the volume of sampled blood is an important quality indicator for blood cultures and should be monitored (3, 148, 149). Blood volume inoculated in a blood culture bottle can be estimated by weighing the bottle before and after sampling, translating the weight to volume by correcting for the density of blood (1.06 g/ml) (150).

However, sampling large volumes of blood is not without risk of iatrogenic anemia in children (151). Moreover, it is not culturally acceptable to patients and healthcare workers in many LMICs (152, 153). Although this has not been widely studied, it is telling that even large clinical studies conducted in Africa using blood cultures sample only one bottle of 5–10 ml of blood in adults (154, 155).

#### Recommendations for Adults

For adults, both the Clinical Laboratories Standards Institute (CLSI) and Cumitech recommend to sample two blood cultures of 20–30 ml of blood each (3, 88) over 24 h, thus adding up to sampled volume of 40–60 ml. One blood culture is defined in these, and most other, guidelines as the volume of blood sampled through one venipuncture (70, 88). These recommendations are based on two older studies using manual blood cultures, where two consecutive blood cultures were found to detect 88 and >99% of BSI episodes (106, 156).

More recent studies suggest that even higher volumes of blood should be cultured. Cockerill et al. found that with an automated blood culture system, 65.1% of BSI episodes were detected with the first blood culture (consisting of 20 ml of blood) (6). By adding a second blood culture, 80.4% of episodes were detected, and up to 95.7% were detected when three blood cultures were used. A study by Lee in 2007 indicated similarly that two blood cultures of 20 ml each in a 24-h period detected 89.7% of BSI (147). In this study, three blood cultures of 20 ml detected 98.2% of BSI and only with four blood cultures, >99% of BSI episodes were detected. Another recent study confirmed the need for at least three blood cultures (or 60 ml) to be sampled, as they found that 7.5% of BSI episodes would have been missed without a third blood culture (157).

A particular situation in terms of number of cultures to sample is suspicion of endocarditis. In this case it is generally recommended to sample three blood cultures of 20 ml, as this has showed to detect 96 to 98% of BSI in the context of endocarditis (158).

In many studies, a distinction is made between optimal volume per blood culture and optimal number of cultures to be sampled per BSI episode. However, as it has been established that drawing blood cultures at intervals or at the time of a fever peak does not contribute to a higher recovery of pathogens (159, 160), the key variable is the total volume of blood that is cultured, irrespective of time and frequency of sampling. For example, if it is recommended to sample 60 ml of blood for culture, it may not make much difference whether this 60 ml is cultured as two blood cultures of 30 ml or as three blood cultures of 20 ml (23, 145, 161).

#### Recommendations for Children

In children, the situation is more complex. The volume of blood that can safely and comfortably be sampled in children is related to the child's age and weight. It was long believed that very small volumes of blood were sufficient for blood culture in children, as bacterial concentrations in children with BSI were thought to be much higher than in adults (162). However, some studies have shown proportions of low-level bacteremia (< 10 CFU/ml) in 23 to 69% of infants and children (163–165). In line with these, sampling higher volumes has been shown to increase sensitivity of blood cultures in children (163, 166, 167). Given the high proportion of low-level bacteremia that was found by Kellogg et al. (163), the authors recommended to sample up to 4–4.5% of the patient's total blood volume.

The latest guidelines from the Infectious Disease Society of America and American Society of Microbiology, recommend sampling 2.5 to 4% of total blood volume from children (74) (Table 4). However, a review from 2011 suggests that only 3.8% of total blood volume can be safely sampled (for all laboratory analyses combined over a 24 h period) in children beyond the neonatal period, and this safety value is further challenged by higher rates of severe anemia in children in LMICs, particularly caused by severe *Plasmodium falciparum* malaria, which predisposes to Gram-negative BSI (43, 151, 168, 169). Given the necessity in many seriously ill children to sample blood for other analyses as well, sampling 4% of total blood volume for blood culture alone can therefore not be recommended in LMIC. More in line with these concerns, CLSI recommends sampling of maximum 1% of the total blood volume (for infants and younger children) (88).

**Table 4:** Recommended volumes of blood for culture in children.

**Weight of patient (kg)**	**Total patient volume (ml)**	**Recommended volume of blood for blood culture (ml)**		**Total volume for culture (ml)**	**Percentage of patient's total blood volume**
		**Culture 1**	**Culture 2**		
≤1	50–99	2	-	2	4
1.1–2	100–200	2	2	4	4
2.1–12.7	>200	4	2	6	3
12.8–36.3	>800	10	10	20	2.5
>36.3	> 2200	20 – 30	20 – 30	40 – 60	1.8 – 2.7

*Based on IDSA Microbiology guideline (74)*.

**Figure 7:**
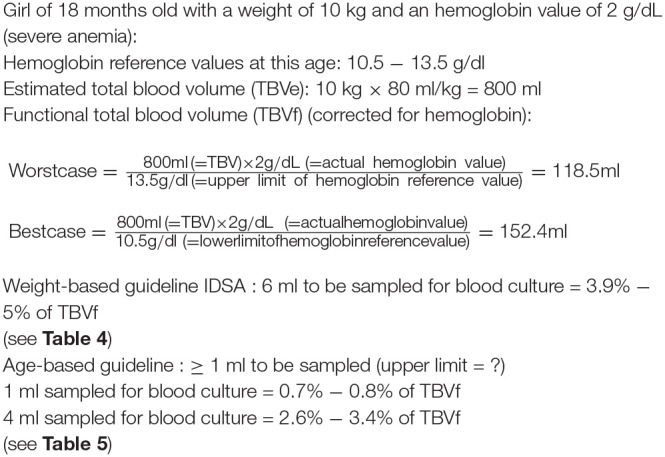
Example of safe sampling in an 18-month old child with severe anemia caused by *Plasmodium falciparum* malaria [formulas used from Kuijpers et al. (169)]. The weight-based guideline recommends sampling of higher volumes than the age-based guideline. This example demonstrates how, in cases of severe anemia, the weight-based guideline may not be safe in children.

Many articles however recommend an age-based sampling strategy illustrated in Table 5 (166, 170, 171). This age-based sampling is convenient and safe, as the volumes are noticeably smaller than with weight-based guidelines (see Figure 7 for an example in LMICs). However, no upper limits are defined for any of the age categories, leaving quite some room for interpretation. For children over 36 months of age, this guideline risks sampling insufficient volume. It is unlikely that the same volume is appropriate for a 4-year old child as for a 13-year old child, but this distinction is not made. Unfortunately, as CLSI remarks, “There are no published data for determining when volumes considered to be appropriate for adults can be used for older children” (88). As was pointed out by Dien Bard et al. in 2016, there is no consensus between different guidelines on which volume to sample in children (172), so controversy remains.

**Table 5:** Recommended blood volumes for blood culture in children based on age (166, 170, 171).

**Age**	**Volume of blood to be sampled**
<1 month	≥0.5 ml
1–36 months	≥1 ml
≥36 months	≥4 ml

#### Conclusions on Volume

In conclusion, it is recommended that for adults at least 40 ml of blood should be drawn, divided over 4 blood culture bottles, to obtain sufficient sensitivity. If feasible, it is recommended to sample an additional 20 to 40 ml of blood. It is unnecessary to sample more than 80 ml of blood, as >99% of pathogens will be detected at this point (147). In case of suspicion of endocarditis, 3 blood cultures of 20 ml each must be sampled. For children, recommendations are less straightforward. Because of simplicity, concerns regarding iatrogenic anemia, widespread use in hospitals and comparability across published literature, we recommend the use of the age-based simple algorithm depicted in Table 5 (e.g., sampling ≥0.5 ml for neonates <1 month of age, ≥1 ml for children age 2–36 months and to sample ≥4 ml in children ≥36 months of age), despite our awareness of the limitations of this strategy.

### Preventing Contamination of Blood Cultures

Sometimes, bacteria not present in the blood of the patient grow in blood cultures after having been introduced into the bottle during broth preparation, blood sampling or processing of the blood sample. These blood culture contaminants usually originate from the environment or the patient's skin (173). Most frequently, contamination happens during venipuncture, when skin fragments containing normal skin flora are dislodged and aspirated together with the sampled blood. Examples of common skin contaminants are coagulase-negative *Staphylococcus* species, *Corynebacterium* species and *Cutibacterium acnes* (formerly *Propionibacterium acnes)* (173). Contamination from the environment is also possible; *Bacillus* species (other than *Bacillus anthracis*) are often seen in this case.

#### Distinction Between a Contaminant and a Pathogen

It can be difficult to make the distinction between a contaminant and a pathogen, as some typical blood culture contaminants such as coagulase-negative *Staphylococcus* species can cause catheter infections or other foreign body infections. The distinction can be made either by clinical assessment (i.e., review of medical records), or by the number of blood cultures that show growth for this particular organism. Often, such an organism is only regarded as clinically relevant if it is isolated in at least 2 separate cultures (and venipunctures), because the odds of having contaminated both cultures with the same pathogen are very small (174). However, this approach cannot be used in settings where only one blood culture is sampled. Time-to-detection can also be of help in the interpretation, as it has been shown that contaminants show slower growth than true pathogens (6, 119, 175).

*Corynebacterium* species, *Bacillus* species (other than *Bacillus anthracis*), *Micrococcus* species, *Lactobacillus* species and *Cutibacterium* species are rarely associated with clinical infections and are almost always contaminants (176). Coagulase-negative staphylococci can cause true infection but are much more often implicated as contaminants. Isolation of *Enterococcus* species, non-fermenting Gram-negative species (e.g., *Acinetobacter* species or *Stenotrophomonas maltophilia*) and viridans streptococci is often of uncertain clinical significance, complicating their role in the interpretation of blood culture results (70, 176).

#### Contamination in LMIC

Contamination of blood cultures remains a substantial problem, with contamination rates as high as 10% even in many HICs (177). Contamination is suspected to be more frequent in LMICs, with some settings (i.e., South Africa, Ghana, the Gambia, Malawi) indeed reporting very high contamination rates (178–181). The spectrum of contaminants in LMICs differs slightly to HICs; for example, more *Bacillus* species are seen in LMICs (178). This finding suggests that contamination of blood cultures in LMICs commonly has the environment as a source, as *Bacillus* species are known to be present in dust and have been described in outbreaks of pseudo-bacteremia originating from the environment (182, 183). More confusingly, some organisms representing “true pathogens” in HICs, such as *Pseudomonas aeruginosa* or other non-fermenting Gram-negative organisms, are sometimes considered as contaminants in LMICs (181, 184).

Laboratory work-up of these contaminants demands time and money, both of which are not in large supply in resource-limited settings. Furthermore, contaminants lead to longer hospital stays, increased prescription of antibiotics and related morbidity (185–188), and may hamper growth of pathogens. Therefore, avoiding contamination is of utmost importance and can lead to substantial cost savings and improvements in patient management. Current guidelines advocate a target of <3% contamination rate and ideally <1% (70, 88, 173). In the following paragraphs, some strategies are described to avoid contamination.

#### Strategies to Reduce Contamination

##### Skin antisepsis

WHO's recommendation from 2010 regarding the type of antiseptic preferably used for blood sampling in the context of blood transfusion is 2% chlorhexidine in 70% isopropyl alcohol (189). No guidance is given regarding blood sampling in the context of blood culture. The CLSI guideline of 2007 on procedures for blood cultures states that tincture of iodine (alcohol-based iodine) and chlorhexidine gluconate are probably equivalent to each other but superior to povidone iodine (water-based iodine) (88). Unlike iodine, chlorhexidine is not associated with allergic reactions, although its safety in infants <2 months of age has not been established (190–192). A lot of evidence suggests that products containing alcohol are better than those without alcohol (193–199), a finding which may be due to the fact that alcohol dries faster than water-based products and therefore less waiting time is required to obtain maximal disinfecting activity. Alcohol has an immediate onset of action, but the effect is not sustained, which is why it is often combined with other antiseptics with a residual effect, such as chlorhexidine (196).

In contrast to this, a recent meta-analysis found no significant difference between any of the antiseptics under study (povidone iodine, iodine tincture, chlorhexidine compounds, or chlorhexidine alcohol) (200), although its methods have been criticized (201). The debate on the most ideal antiseptic is thus ongoing, but most antiseptics seem to give good results when used properly. For this reason, alcohol-based antiseptics may be more effective, as the drying time is shorter, and they are therefore more likely to be used correctly. In addition, alcohol-based solutions are less vulnerable to accidental colonization with Gram-negative bacteria compared to water-based antiseptics (202).

##### One-step vs. two-step procedure

The comparison of one-step (single application of an antiseptic) vs. two-step procedures (consecutive application of the same or different antiseptics) for skin antisepsis has been made mainly in the context of blood collection for blood donation. These studies generally evaluate the effect of antiseptics on the bacterial load of the skin (203). A Cochrane review of 2015, however, found no experimental or quasi-experimental publications which had studied the effect of one-step vs. two-step procedures on actual blood culture contamination (204).

Therefore, no recommendation can be given regarding the effectiveness of either procedure. In many LMICs, patients have had long travels on dusty roads before reaching the hospital. It is therefore good practice to use a two-step procedure for antisepsis: the first application of antiseptic (preferably isopropyl alcohol 70%) can be used to clean the skin of dust and dirt. Cleaning with alcohol swabs must be repeated until the swabs are visually clean—since, unlike ethanol, isopropyl alcohol has a cleaning effect (205). Only when the skin is clean, the second step of antisepsis can be performed.

##### Phlebotomy teams

To further decrease contamination, there is quite good evidence for the use of dedicated phlebotomy teams for blood culture, as opposed to blood cultures being sampled by all ward nurses or interns (193, 197, 206, 207). This approach has also had good results in a low-income country; after the introduction of a team of nurses dedicated to phlebotomy in Malawi, contamination rates decreased from 19.6 to 5% (179).

##### Blood culture collection packs

Another possible strategy is the use of blood culture collection packs. Blood culture collection packs are prepackaged kits assembling all materials needed for blood culture. Although many studies have reported lower contamination rates after the introduction of blood culture collection packs (208–210), a meta-analysis of 2012 showed no conclusive evidence for this practice (207). Most of the publications which have reported a decrease in contamination rates combined introduction of the blood culture collection packs with hospital staff training and awareness campaigns, thereby compromising validity of the research. Given the added cost of blood culture collection packs, more evidence is needed before its implementation can be recommended in LMICs (207).

##### Diversion of first portion of blood

Contamination of blood samples can occur during venipuncture because of dislodging of skin fragments colonized with bacteria. Diverting or discarding the first few milliliters of blood can possibly decrease contamination due to this cause. The first study done to evaluate this method was the trial by Patton and Schmitt (211), which indeed showed decreased contamination after diversion of the first ml of blood into a sterile Vacutainer® tube (Becton-Dickinson Diagnostics, Franklin Lakes, NJ, USA). Contamination went down from 2.8 to 1.4%. This concept was repeated by Binkhamis et al. (212) and Rupp et al. (213); both studies found significant results with a reduction in contamination rates of 3.4–2.4% and 1.78–0.22%, respectively. In all three studies, pre-intervention contamination rates were already low. In LMICs with higher contamination rates, the same relative reduction could potentially lead to a large decrease in contaminated samples.

Usually, it is advised to sample first blood cultures and only next the tubes for other blood analyses. This recommendation is based on the observation that reflux from contaminated citrate- or EDTA tubes to the syringe may occur, with possible contamination of the blood culture sample (177, 214–216). However, these reports of “pseudo-bacteremia” caused by contaminated blood collection tubes all date from the 1970's and 1980's and the current risk for this is unknown. Given the effectiveness of diverting the first portion of blood, contamination could actually be reduced by sampling first other blood collection tubes if a butterfly needle with vacutainer system is used. Sterilization of blood collection tubes may further prevent cross-contamination.

##### Multi-sampling vs. single-sampling strategy

The CLSI guideline recommends collection of two to three blood cultures, each consisting of two blood culture bottles sampled through one venipuncture, per episode (88). However, it has been shown that a time interval between the different draws is unnecessary (159, 160). Therefore, the necessity of sampling blood using separate venipunctures, referred to as multi-sampling, has been contested. There are obvious practical advantages to sampling blood by just one venipuncture, referred to as single-sampling (23). This single-sampling approach may also theoretically decrease the risk of contamination (161, 217). Unfortunately, few studies directly comparing both strategies are available. One trial in France showed increased positivity rate, improvement of overall performance (sensitivity and specificity) and better compliance to protocol of the multi-sampling strategy compared with the single-sampling strategy (218).

Contamination, however, can be more difficult to judge when single-sampling instead of multi-sampling is used. The reason for this is that many low-virulence organisms are no longer considered as contaminants when they are isolated from blood cultures sampled through more than one venipuncture (69, 88, 174). However, an evaluation by Arendrup et al. did not find the interpretation to be more difficult when using the single-sampling strategy (219). They also demonstrated that the recovery of pathogens was correlated with the number of grown blood culture bottles, suggesting this indicator can be used to determine the presence of contaminants.

The recommended approach will probably depend on the setting and patient population; in a setting with very few central venous catheters or prosthetic devices, it is probably not needed to sample from different venipuncture sites, because infections with organisms of low virulence are very unlikely in this setting. In many LMICs there is a reluctance to sampling blood in both staff and patients (1), so a single-sampling strategy will probably more acceptable. In settings where the interpretation of possible contaminants is more difficult, it is still recommended to sample from at least two separate venipunctures.

##### Sampling through catheters

Many studies have shown increased contamination rates when blood is sampled through intravascular catheters, as opposed to venous puncture (177). However, most of this evidence is found when using central lines for blood sampling. This suggests that sampling blood when placing a peripheral intravascular catheter might be safe and practical, especially in children in whom a reduction of the number of venipunctures is desirable. Indeed, some studies did not show a higher contamination rate from blood sampled through a recently placed peripheral catheter than through separate venipuncture (220–223). However, many other studies have found higher contamination rates with this practice (224–227). Hall et al. described contamination rates as low as 1.6% when sampling via newly inserted catheters by following a sterile technique protocol in a pediatric emergency department (228). In general, sampling through peripheral catheters must be discouraged, but its practical advantages cannot be denied and should be balanced against the risk of increased contamination.

##### Disinfecting the blood culture bottle septum

Another measure to decrease contamination is to disinfect the blood culture bottle septum with 70% isopropyl alcohol or ethanol before injecting the blood sample (88, 193, 229, 230); in many institutes, however, this is not done routinely (193, 231). Bekeris et al. did not find evidence for the effectiveness of this measure in decreasing contamination, however they noted that their study was not powered to detect such an effect as they had quite low rates of contamination overall (206). In the context of higher environmental contamination in LMICs, disinfection of the blood culture bottle septum is an easy and low-cost strategy to avoid contamination.

##### Sterile gloves

Sterile gloves may reduce contamination rate but increase the cost of blood cultures (232, 233). Whether this incremental cost will be offset by decreased costs associated with work-up of contaminants will need to be evaluated and will depend on the baseline contamination rate (177). As there are many inexpensive ways to decrease contamination, sterile gloving might not be the most cost-effective option when looking for a strategy to reduce contamination rates. In most studies evaluating the use of sterile gloving, the absolute reductions in contamination rates following its implementation were low, leading to a high cost for very few averted contaminants (232, 233). However, baseline contamination rates were also low in these studies; in settings where contamination is much more prevalent, such as most LMICs, the same relative decrease in contamination rates will lead to a higher number of averted contaminants. In that case, the intervention will have a better cost-benefit ratio.

##### Collection system

Although no impact on contamination rates has been described to our knowledge, the choice of the type of collection system will be briefly discussed in this paragraph. For collection of blood cultures, either a butterfly needle with Vacutainer® system or a simple needle with syringe method can be used. It is not possible to increase the safety of the needle/syringe system by using safety devices (such as retractable needles), as the healthcare worker will still use the needle after sampling to inoculate the blood in the blood culture bottle. The time between sampling and inoculation into the bottle is the period of risk of needle stick injury (234). It is also not recommended to connect a blood culture bottle directly to the needle using a Vacutainer® system, as backflow of the broth into the patients' veins may occur. Butterfly needles on the other hand, are easy to use, especially when other blood analyses must be done as well; and other blood collection tubes can be connected to the Vacutainer® system after the blood culture bottles have been filled without the need for a separate venipuncture. Additionally, they are probably safer to use than needle-and-syringe as connection to the bottle is easier (provided the correct adaptor) (234). However, they are more expensive. If financially sustainable, butterfly needles are therefore recommended.

Lastly, changing needles between taking blood cultures and inoculating the blood into the blood culture bottle (in case of syringe and needle procedure) may decrease contamination rates slightly (235); however, it also increases the risk of needle stick injury and it is therefore not recommended (88, 189).

### Monitoring of Quality Indicators

Unlike for other specimens, such as urine and respiratory secretions, many quality indicators which are useful to assess, and monitor have been described for blood cultures. They can be used to improve the processes from request to report in the hospital and laboratory by giving directed feedback to the staff involved. Examples of quality indicators are given in Table 6. Although many of these indicators seem clear-cut at first sight, definitions are not always uniform, and this leads to varying rates and figures being reported.

**Table 6:** Quality indicators for monitoring of blood cultures—they can be used for validation as well as for monitoring purposes.

**Quality indicator**	**Definition**	**Goal**	**Comment**
Proportion of blood cultures that show growth with a pathogen (positivity rate)	Number of blood cultures showing growth with a pathogen /total number of blood cultures	5–15% (3) 6–12% (70) If lower than the goal, ordering of blood cultures is too liberal; if higher than the goal, it is too stringent	These figures are appropriate for HICs and settings where malaria is not endemic. Studies performed in LMICs often show higher proportions of pathogens (19).
Total number of blood cultures	Number of blood cultures/1000 patient days	103–188 (3)	Has exclusively been studied in HICs; goals for LMICs not clearly defined
Missed opportunities	Number of missed opportunities for blood culture sampling as assessed by patient file review (236)	Not defined	
Contamination rate	Number of contaminated blood cultures/total number of blood cultures	<3% (3, 70) <1% (70)	For this definition, blood culture is defined as blood sampled through one venipuncture
Volume	Volume per blood culture bottle	≥80% of recommended volume (3, 70, 88)	Following formula can be used (the factor 0.94 expresses the correction for density of blood): Volume per bottle = (weight of bottle after sampling – weight of bottle before sampling + average weight of cap) ^*^ 0.94
Number and proportion of solitary blood cultures	Adult blood cultures consisting of only one blood culture bottle instead of at least two	Best performing hospitals have only 3.4% solitary blood cultures (3)	
Needle-to-incubator time	Time interval from blood culture sampling to incubation	<2 h (88) <4 h (70)	
Time-to-detection of growth	Time interval from incubation to detection of growth	Not clearly defined for manual blood culture bottles	
Gram stain accuracy	Correlation between smear result (Gram stain) and culture result.		
Turnaround time	Time interval from registration of the sample in the laboratory to reporting of the result to the clinician (237)		
Quality of antibiotic susceptibility testing (AST) report	Correct interpretation and reporting of raw results		

For example, contamination rate will vary across studies depending on how contamination has been defined and calculated, and often this is not well-described. According to Leber, the contamination rate is calculated “by dividing the number of cultures containing contaminants by the total number of cultures collected by venipuncture,” with a blood culture being defined as “blood from a single venipuncture” (70). This suggests that blood culture contamination rates are defined as the contamination per venipuncture (and not per blood culture bottle), and it is thus important to know how the bottles were sampled exactly. Another problem with contamination rates is the definition of contamination itself, which may not be uniform across studies. Depending on the study, the species involved in contaminated blood cultures may differ, the number of cultures growing the contaminant may or may not be taken into account, or clinical review of the patient files may or may not have been done to determine true contamination. It is therefore important to assess the definition used in the study when comparing contamination rates.

## Challenges

Although we have argued in paragraph 1.3 that blood cultures are feasible in LMICs, some significant challenges remain. First and foremost, clinical bacteriology facilities in LMICs have to be strengthened and supported. As has been argued elsewhere, problems of staff training and retention, insufficient infrastructure, and lack of stringent quality control and equipment maintenance jeopardize all laboratory activities, including blood cultures (1, 2, 153). Other challenges, specific to blood cultures, will be discussed in the paragraphs below.

### Detection of Growth

Manual blood culture systems require inspection of the blood culture bottles once or twice daily. Most signs of growth are rather subtle, and experience and training are therefore needed to recognize growth in the bottles. Manipulation of blood culture bottles can result in stirring the blood cells with the broth, which further complicates the detection of turbidity. Standardized visual conditions are preferable for reliable detection of growth. This includes standardized backgrounds and lighting; normal daylight may show substantial variation; therefore, the use of a lightbox can be considered.

#### Blind Subcultures

Because of the challenges related to visual detection of growth, other strategies were employed before the widespread use of automated monitoring of blood culture bottles. For manual systems, “blind subcultures” are often recommended. These are subcultures from the blood culture broth on agar plates, in the absence of visual signs of growth. The optimal timing of this subculture varies according to the consulted source. Depending on the moment of blind subculture during the incubation [for instance early at day 1 vs. at day 7 at the end of the incubation period (so-called “terminal” subculture)], blind subculture can be used either as a way to shorten the time-to-detection or as a final check for growth.

CLSI for example recommends subculture after 24 to 48 h of incubation to facilitate early detection of microorganisms (88). The American Society of Microbiology “Clinical Microbiology Procedures Handbook” by Leber et al. (70) recommends blind subculture at 72 h. Cumitech advises routine blind subcultures after 12–18 h of incubation for aerobic bottles (3, 70). Routine blind subcultures are not advised for anaerobic bottles (3).

Early subculture (within the first 24 h of incubation) seems to be a successful strategy for rapid detection indeed. Szymczak et al. detected 85% of pathogens with blind subculture within 24 h, whereas in only 48% of positive cultures visual signs of growth could be detected within 24 h of incubation (238). Even earlier subculture, within four to 14 h after incubation, led to detection of 50% (239) and up to 85% of pathogens (240). Studies performing subculture between 6 and 17 h after incubation detected 48% (241) and 63% (242) of pathogens. Subcultures performed within 6 h yielded only 10% of pathogens according to the study by Sliva et al. they therefore do not recommend subculture within 6 h of incubation (242). A drawback of blind subcultures is that by opening the bottle and introducing a syringe or pipette, contamination may be introduced.

#### Terminal Subcultures

Terminal subcultures (blind subcultures of blood culture bottles at the end of the incubation period) are considered unnecessary by many and may increase contamination rates and needle stick injuries (243–245). However, they may still be considered in systems which do not perform a routine blind subculture at an earlier time or in situations with prolonged incubation (245). They are also useful at the validation phase of a newly introduced blood culture system. In settings with many immunocompromised patients, where higher numbers of *Pseudomonas aeruginosa* and yeast BSI are anticipated, terminal subculture could also be of value, as these organisms tend to show slower growth and more subtle visual signs of growth, presumably due to smaller initial concentrations of bacteria (246).

### The Need for Speed: How to Decrease the Time-to-Detection and Turnaround Time

It is well-known that automated blood cultures have a much shorter time-to-detection than manual systems. For manual systems, cumulative growth within 48 h of incubation varies between 65.8 and 94% (137, 238, 240, 241, 247, 248). It must be noted that all of these studies performed a blind subculture within 24 h of incubation; not performing blind subculture may even lead to lower detection rates after 48 h of incubation. This is obviously longer than what is currently seen with automated systems, where 82–91.6% of growth is detected within 24 h (249–251). Some recent studies from LMICs, directly comparing manual with automated systems, also found higher sensitivity, specificity and especially much lower time-to-detection with the automated system (24–26).

The importance of reducing the time-to-detection of blood cultures cannot be overestimated, for both antibiotic stewardship purposes and to increase demand for blood cultures by clinicians in LMICs, as patients are often discharged or leave the hospital against medical advice early in the course of treatment (252, 253). Long turnaround times of test results are a common complaint in LMICs, decreasing the trust in the laboratory services (254, 255). Moreover, rapid communication of preliminary results, such as the Gram stain, is often not or insufficiently done in LMICs (153). Effective and immediate communication of blood culture preliminary results, ideally combined with clinical advice, should be a priority for bacteriology laboratories (312, 313).

#### Agitation

It may be impossible to increase the speed of detection of manual systems up to the performance of the automated systems, as the algorithm-driven continuous monitoring will prove difficult to mimic in an equipment-free manual system. However, another feature of automated systems is continuous agitation during incubation. Agitation is thought to improve microbial recovery by increasing the oxygen concentration in the broth medium and will therefore speed up detection of aerobic bacteria (69, 256–258). The “Clinical Microbiology Procedures Handbook” recommends continuous agitation of manual systems as well (70). Continuous agitation, however, impacts ease of visual growth detection, as turbidity will increase due to mixture with the red blood cells.

#### Venting

Another strategy to speed up growth is venting, i.e., inserting a filtered needle or other device that allows air to enter into the bottle headspace. The need for venting of aerobic blood culture bottle depends mainly on the manufacturer of the blood culture bottle. For automated systems, it is agreed that venting is not necessary (69, 259), but some commercial manual blood culture bottles may still need venting. Most studies evaluating the impact of venting stress the role of appropriate amounts of oxygen; this seems especially important for *Pseudomonas, Candida* and other strictly aerobic species, both for rate and speed of growth (77, 144, 241, 260).

It is currently unclear whether venting is still required with the newer commercial manual bottles; there are no recent studies on this. Of course, venting involves an extra procedure to be carried out, thereby potentially contaminating the sample. Despite this theoretical possibility, there is no documented evidence for increased contamination with vented bottles.

#### Laboratory Organization

Clinical importance of increased speed of detection is also influenced by laboratory organization. Notification of the Gram stain result to the clinician has been shown to have the highest clinical impact (56, 58, 59). Laboratories not providing 24-h service will probably see less benefit from small differences in time-to-detection, as they may be unable to translate this into quicker results to the clinician. Furthermore, the most important actionable result is growth in the first bottle of a BSI episode; growth in the other bottles of that episode are of lower clinical impact. When taking all this in consideration, the clinical relevance of difference in speed between automated and manual systems may be lower than expected. Future studies comparing speed of growth of different systems, including comparisons between automated and manual systems, should take these considerations into account.

A study that has looked at the practical organization in the lab was performed by Youngs et al. in 1985 (261). They recognized the added value of early subculture (after 6–17 h of incubation) but mentioned that the actual timing of subculture also depended on arrival of the bottle in the lab. To be sure that a subculture was performed within 6–17 h, they implemented two timeslots for subculturing: one in the early morning for all bottles having arrived after office hours the previous day and another subculture at the end of the work day for all bottles that arrived during the day. This led to growth of pathogens on solid media the day after incubation for 10 out of 14 grown blood cultures, making an appreciable impact for patient management, as none of these bottles had shown visual signs of growth the day before (261).

#### Transport to the Laboratory

A relatively simple way of improving the speed of detection is by ensuring that the bottles arrive in the laboratory in a timely manner. It has been shown that a delayed incubation significantly prolongs detection time and may lead to false-negative results in automated systems (23, 262, 263), however the exact critical time has not been formally established. Guidelines recommend maximum needle-to-incubator time of 2 h (88) to 4 h (70); however, many centers actually observed much longer average transport times (264). Long distances and irregular transport of laboratory specimens from the site of collection to the laboratory is more common in LMICs, resulting in even higher transport times.

Blood culture bottles must be transported to the lab at room temperature (88, 265) or in a thermostable carrier and should never be refrigerated or frozen, as many fastidious organisms are vulnerable to cold (88, 266). Pre-incubation of blood culture bottles at 35°C, for example in a small incubator in the emergency department, can speed up detection of growth in manual blood culture systems (267), but is not advised for automated systems as it may result in false-negative results (23, 268–270). However, high “room temperatures” are common in LMICs, warranting short transport times when using automated blood culture systems to avoid false-negative results.

#### Direct Testing on Blood Culture Broth

Performing direct identification and/or antibiotic susceptibility testing on grown blood culture broth can also lead to faster diagnosis and decision-making. Molecular assays and matrix-assisted laser desorption/ionization—time-of-flight (MALDI-TOF) methods are currently out of reach for most LMICs, but lateral-flow immunoassays and other simple testing methods can provide a convenient solution. A number of simple direct tests have been described. Examples are detection of *Staphylococcus aureus* by direct tube coagulase test for detection of *Staphylococcus aureus* (271, 272), latex agglutination tests (273) and immunochromatographic tests (274), and the recent development of lateral flow assays for *Burkholderia pseudomallei* (275) and *Salmonella* species (276).

For antibiotic susceptibility testing, lateral flow assays have been developed to rapidly detect methicillin-resistant *Staphylococcus aureus* (277), extended-spectrum beta-lactamase (ESBL) (278) and carbapenemases (279), directly from blood culture broth. Moreover, the European Committee on Antibiotic Susceptibility Testing (EUCAST) recently provided guidelines on direct disk diffusion testing from blood culture broth, with adapted procedure and diameter cut-offs^4^. Performing antibiotic susceptibility testing directly from blood culture broth decreases the turnaround time with 1 day but requires rapid identification as the procedure often depends on the pathogen.

### Production, Distribution, and Procurement of Blood Culture Media

#### Accessibility and Affordability of Blood Culture

Access to quality-assured *in-vitro* diagnostics is a problem in LMICs; diagnostics are frequently more expensive when purchased in a LMIC and they are not always easily available (104, 280, 281). Although data are rare, it is estimated that the cost of blood cultures is twice as high in LMICs than HICs (41). Most healthcare systems in LMICs rely heavily on out-of-pocket payments of patients, including for diagnostics (153, 282, 283). According to a recent WHO report, out-of-pocket payments, although in decline, still account for 37% of the total healthcare cost in Africa and 44% in South-East Asia (282). Assuming a positivity rate of only 10% for blood cultures, the cost-benefit ratio for the individual patient of performing blood culture may be unacceptable in very poor populations. A Kenyan study of 2009 reports a blood culture cost of 18.36 US $ per individual test, much higher than other diagnostic tests in the same setting such as HIV test (3.68 US $) or malaria microscopy (3.50 US $) (284). Moreover, this price refers to a single blood culture bottle; as mentioned before, often two to four blood culture bottles are needed per patient, further increasing the total price. According to a panel of stakeholders, an acceptable cost for blood cultures would have to be <10 US $, and ideally <5 US $ (153). In the short term, donor involvement will therefore be necessary for blood cultures to be affordable.

Transport of commercial blood culture bottles from abroad is very costly and transport times are considerable, compromising shelf life. Low volumes of cultures per lab, skilled labor needs and short shelf lives further affect affordability of blood cultures in many settings (41). For this reason, many laboratories resort to in-house prepared blood culture bottles, typically containing brain-heart infusion or trypticase soy broth (41). This practice is not advised, because the origin and quality of the culture media are difficult to track down and useful additives, such as the ones described in Table 3, will not be present in the broth, compromising performance. An alternative to the commercially available bottles is to support local production of blood culture media by reference laboratories, from where they can be dispersed to smaller hospitals (2, 285). An exemplary initiative is the laboratory logistic and educational support provided by the Diagnostic Microbiology Development Program in Cambodia (1)^5^.

#### Need for Anaerobic Culture

When choosing a blood culture medium to procure, it is good to remind that there is no perfect all-purpose blood culture medium. The ideal medium will depend on the expected pathogen distribution, which may vary according to the setting. Furthermore, the choice of broths will depend on whether anaerobic blood culture is deemed necessary. Most centers that analyzed the frequency of isolation of anaerobes from blood cultures have noted a declining incidence; coinciding with this, other (aerobic) organisms such as fungi, *Pseudomonas* species and fastidious aerobic bacteria are increasingly associated with disease (69). Some have suggested to replace routine inoculation of anaerobic media by a second aerobic bottle and reserve the anaerobic bottle only when clinically indicated or in patients at risk of anaerobic infections (286–289).

Not all hospitals, however, have seen this decrease in anaerobic bacteremia (290, 291). Moreover, Vena et al. found that in half of the cases of anaerobic infection, this could not have been suspected on clinical grounds (291). The choice of which bottles to use is therefore highly dependent on the specific setting, and every center is encouraged to perform its own evaluation on the need for anaerobic blood culture (69). These differences in regional incidence of anaerobic isolates from blood culture were also confirmed in a more recent review (292). In this review, it is stated that anaerobic cultures can safely be omitted in most cases, unless clinical indications for anaerobic infections are present. Moreover, identification and antibiotic susceptibility testing of anaerobic bacteria is often difficult in LMICs due to the need of specialized and generally more expensive techniques^6^ (293). CLSI however still recommends that routine blood cultures include paired aerobic/anaerobic blood culture bottles, as the data are conflicting and the recommendation of limiting anaerobic blood culture bottles has never been validated by controlled clinical studies (88, 290, 294, 295). Because anaerobic bacteria are rarely recovered in pediatric patients, and usually only one bottle is sampled, the use of only aerobic bottles has been recommended in this patient population (296, 297).

#### Physical Properties of the Bottle

**Figure 8:**
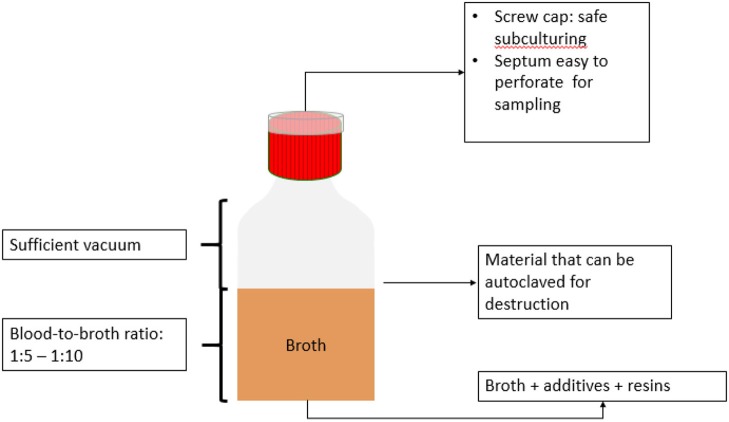
Dissection of blood culture bottle.

Equally important is the selection of the correct bottle type (Figure 8); for reasons of (bio)safety and shipping costs, plastic bottles may be preferred over glass bottles. The specific type of plastic may be important for subsequent waste management (see below). The bottle material also needs to be completely transparent to allow visual inspection for growth. Bottles have to be stored and shipped adequately to avoid scratches on the material. To increase ease of use, the septum of the bottle must be easy to perforate by a needle and the vacuum in the bottle must be sufficient to aspirate the required amount of blood (10 ml for adult bottles, 4 ml for pediatric bottles). A screw cap may be more practical than other cap types in terms of waste management; after autoclavation, the fluid can then be poured out safely and the empty bottle incinerated. A screw cap is also advantageous for sampling the blood-broth mixture (e.g., for subculture), as this can avoid the use of needles.

#### Tropicalization of Blood Culture Bottles

Lastly, blood culture bottles should be “tropicalized”, i.e., adapted to harsh environmental conditions (1, 153), in order to be useful for LMICs. In many LMICs, no manufacturers of *in-vitro* diagnostics are locally present, necessitating import and shipment from other countries. Therefore, shipment stability is an important factor, as is a sufficiently long shelf life. Many compounds in blood culture bottles, such as resins and SPS, may shorten shelf life, as exemplified by the much shorter shelf life of bottles designed for automates when compared to manual blood culture bottles. In line with requirements for malaria rapid diagnostic tests according to the Prequalification of *in vitro* Diagnostics (PQDx) Programme^7^ of the WHO, shelf life must be at least between 1.5 and 2 years. Storage temperature must be sufficiently flexible to allow storage or at least shipment at tropical temperatures (153).

### Signal Without Growth on Subculture

In some cases, blood culture bottles appear “positive” (detection of bacterial growth) by a visual sign of growth such as turbidity or color change of a carbon dioxide indicator, but sub-culturing on solid media does not confirm the presence of bacteria. This phenomenon has mainly been studied for automated blood culture systems, where it can be caused by overfilling of blood culture bottles, elevated blood leukocyte counts, or antibiotic consumption by the patients before blood culture sampling (298–300). Microbiological causes are growth of *Plasmodium falciparum* in the blood culture bottle (301, 302) or growth of fastidious bacteria such as *Streptococcus pneumoniae*, which can exhibit rapid autolysis (i.e., self-destruction) in case of stationary growth, making it undetectable at Gram stain and subculture (303, 304). Other fastidious organisms, such as *Campylobacter* and *Mycoplasma*, can cause turbidity without growth, as they are difficult to stain and do not grow well on agar plates in normal conditions (266).

### Biosafety Concerns in Blood Cultures

#### Need for a Biosafety Cabinet?

A laboratory performing blood cultures usually functions as a Biosafety Level (BSL)-2 laboratory (305). When handling blood cultures, it is possible that laboratory staff comes into contact with unknown pathogens causing airborne infection. This raises the question whether laboratories in LMICs must also be equipped with a biosafety cabinet, keeping in mind the challenges in LMICs of dusty environments, lack of electricity and equipment maintenance (Table 7). A class II biosafety cabinet provides protection to laboratory staff against infectious materials as well as to biological samples against external contamination. The presence of a biosafety cabinet is generally recommended for a BSL-2 laboratory in case of likely aerosol formation or splashes (305, 306, 309). A biosafety cabinet most obviously provides protection from inhalation of infectious aerosols. For most other types of exposure, on the other hand, good personal protective equipment, such as safety goggles, face shield, gloves and masks, and the use of safe and appropriate techniques (no mouth pipetting, use of disposable loops) are sufficient. Therefore, the decision of the installation of a biosafety cabinet in a specific setting should be taken after a risk analysis, taking into account possible laboratory-acquired infections when processing blood cultures, and existing alternative methods to mitigate this risk (Table 7).

**Table 7:** Benefits, risks and limitations of a biosafety cabinet in low-resource settings.

**Benefits of biosafety cabinet**	**Risks/limitations of biosafety cabinet**
Presence of a biosafety cabinet in biosafety level-2 laboratory is considered desirable according to WHO guidelines (305).	Poor location, room air currents, decreased airflow, leaking filters, raised sashes, crowded work surfaces, and poor user technique compromise the containment capability of a biosafety cabinet (306). In those conditions, the biosafety cabinet offers a false sense of safety.
Procedures with a potential of generating infectious aerosols or high splash potential should be conducted within a biosafety cabinet (305, 306). These may include pipetting, mixing, centrifuging, grinding, vortexing, shaking, opening of containers of infectious material with internal pressure that may be different from the ambient pressure such as blood culture bottles (306).	With good microbiological techniques and appropriate and consistent use of personal protective equipment (safety goggles, face shield, gloves, mask), biosafety level-2 agents can be used safely in activities conducted on the open bench, provided the potential for producing splashes or aerosols is low (306).
Pathogens of risk groups 3 and 4 are seen more frequently in low-resource settings as opposed to high-resource settings, so biosafety procedures are likely to be more important.	Workers using biosafety cabinet must be specifically trained for the use of a biosafety cabinet (305, 306). In low-resource settings, skilled workers are often not available, training options are few and trained staff is difficult to retain (307). Biosafety level-3 and 4 require additional training of the staff. A biosafety cabinet Class II is fit for biosafety level-3 purposes but not for biosafety level-4 (305).
In case of an unexpected epidemic with a risk group 3 pathogen, having a biosafety cabinet on site can be used for outbreak investigation or other diagnostic purposes during the outbreak.	The biosafety cabinet must be certified when installed, whenever it is moved and then annually. In many low-resource regions, a number of practical problems prevent this from happening, most notably lack of awareness of this requirement, insufficient resources for maintenance and an absence of local competent, qualified certifiers (307, 308). In a survey of biosafety level-2 and 3 laboratories in 7 countries in the Asia-Pacific region, 30% of biosafety cabinets tested were poorly designed, incorrectly installed, not certified, or operated improperly (308)
The use of pre-filters below the work bench in the biosafety cabinet or the use of door dust filters at the entrance of the laboratory can reduce the dust level within the laboratory and prolong the lifespan of the HEPA filters.	HEPA filters need to be replaced more frequently when working in dusty/dirty environments; replacing HEPA filters has to be done by a trained technician. In regions with low accessibility and no local manufacturers, this may be difficult to do on a regular basis.
	The price of a Class II biosafety cabinet is around 10,000 euro; certifying the biosafety cabinet (which should happen at installation, annually and whenever the device is moved) costs an estimated 1,000 euro in Europe; costs in Africa or Asia are likely to be higher because of much higher transport costs, if available at all.

Because of gas production and possible aerosol formation, opening the blood culture bottle to perform subculture substitutes a risk to the laboratory technician. Apart from measures implemented to protect the laboratory technician from this aerosol, aspects of the bottle itself can play a role in safety. For example, biphasic bottles provide opportunities of subculturing without having to open the bottle. Bottle septa that are difficult to perforate may be a risk for needle stick injuries.

#### Waste Management

Another important biosafety aspect is the possibility to safely destroy (decontaminate) grown blood culture bottles, for instance by autoclaving and incineration. The bacterial load of grown blood culture bottles is very high, easily reaching 10^6^-10^9^ CFU/ml (310). In case of steam autoclavation of blood culture bottles, it must be kept in mind that the fluid inside the blood culture bottle must reach 121°C during at least 15 min to ensure effective decontamination. This requires longer cycles when a higher volume of liquids (e.g., more blood culture bottles) are autoclaved, a phenomenon that is often not taken into account. Monitoring the autoclave cycle by using chemical indicators is often not sufficient, as they will only show the presence of steam but do not give an indication of the temperature inside the liquids (311). The autoclave must be validated before taken into use with the help of temperature sensors (if available) and biological indicators, preferably placed inside (non-inoculated) blood culture bottles. Given the importance of the total volume of liquids in the autoclave on the time needed to sterilize, it is important not to overfill the autoclave with liquid waste and to validate the maximum liquid load the autoclave can still safely sterilize.

Moreover, not all plastic can be safely autoclaved after use. Even if re-use of the bottles is not necessary, it is important that the bottle material can be autoclaved without posing direct biological risks by tearing and implosion of the bottles or loosening of the stopper. Polycarbonate (PC) and polypropylene (PP) bottles withstand autoclavation, however polyethylene terephthalate (PET) bottles cannot be autoclaved without substantial damage to the bottle, potentially causing spill of infectious material inside the autoclave. In case of interrupted or only partially successful cycles, this may bring the healthcare worker into contact with infectious aerosols. Therefore, other types of plastic that withstand autoclavation are preferred (like PP or PC)^8^.

### Future Research Needs

Since the introduction of automated equipment for blood cultures, hardly any further research on manual blood culture bottles and systems has been done. This is all the more surprising given the large market share represented by manual blood culture consumables (almost double that of automated systems) and the projected substantial growth of the market for manual blood cultures in LMICs for the decades to come (27). New research and innovation should therefore also include manual blood culture methods, as we have argued elsewhere (1). The recent Unitaid Fever Diagnostic Landscape points out that short reagent shelf lives, supply chain difficulties and highly skilled labor needs drive up the cost of blood cultures in LMICs (41). As mentioned before, high cost decreases demand in these settings, resulting in low numbers of blood cultures performed. The low numbers then further contribute to higher prices per test. Moreover, the lack of critical testing volume will deny the laboratory technicians the opportunity to gain sufficient experience in processing blood cultures, thus impacting the quality of the test results and decreasing clinicians' confidence in the testing. This will in turn further drive down demand for the tests, resulting in a vicious cycle.

To break this cycle and achieve successful implementation of blood cultures in LMICs, affordable and tropicalized blood culture methods are needed. Research into these methods and close collaboration between academia, stakeholders and product developers is mandatory (1, 153).

## Author Contributions

SO did the literature review, writing of the initial draft and revisions, figure design, and project administration. BB contributed to literature review, revisions and provided critical review. DA, JR, PL, and OL provided critical review and commentaries. JJ and LH had the rationale for this work, contributed to literature review, and supervised manuscript revisions. LH additionally contributed to figure design. JJ additionally contributed to photography of the blood culture bottles.

### Conflict of Interest Statement

The authors declare that the research was conducted in the absence of any commercial or financial relationships that could be construed as a potential conflict of interest.

## Footnotes

^1^https://blogs.worldbank.org/opendata/new-country-classifications-income-level-2018-2019

^2^https://www.who.int/gho/malaria/epidemic/deaths/en/

^3^https://www.bd.com/resource.aspx?IDX=11265

^4^http://www.eucast.org/rapid_ast_in_blood_cultures/

^5^http://dmdp.org

^6^http://www.eucast.org/fileadmin/src/media/PDFs/EUCAST_files/Breakpoint_tables/v_9.0_Breakpoint_Tables.pdf

^7^https://www.who.int/diagnostics_laboratory/evaluations/en/

^8^https://www.mynewlab.com/blog/a-guide-to-autoclaving-plastics-and-glass/
